# Investigation of a Cluster of *Candida albicans* Invasive Candidiasis in a Neonatal Intensive Care Unit by Pulsed-Field Gel Electrophoresis

**DOI:** 10.1100/2012/138989

**Published:** 2012-04-01

**Authors:** Jihene Ben Abdeljelil, Fatma Saghrouni, Imene Khammari, Soukeina Gheith, Akila Fathallah, Moncef Ben Said, Jalel Boukadida

**Affiliations:** ^1^UR02SP13 Research Unit, Ministry of Public Health, Tunisia; ^2^Laboratory of Parasitology-Mycology, Farhat Hached University Hospital Sousse, Sousse 4000, Tunisia

## Abstract

Nosocomial invasive candidiasis (IC) has emerged as a major problem in neonatal intensive care units (NICUs). We investigated herein the temporal clustering of six cases of neonatal IC due to *Candida albicans* in an NICU. Eighteen isolates obtained from the six neonates and two isolates from two health care workers (HCWs) working at the same unit and suffering from fingers' onychomycosis were genotyped by electrophoretic karyotyping (EK) and restriction endonuclease analysis of genomic DNA by using Sfi I (PFGE-Sfi I). PFGE-Sfi I was more effective in discriminating between temporally related isolates. It showed that (i) both HCWs had specific strains excluding them as a source of infections in neonates. (ii) Isolates collected from three neonates were identical providing evidence of their clonal origin and the occurrence of a horizontal transmission of *C. albicans* in the unit. (iii) The three remaining neonates had specific strains confirming that the IC cases were coincidental. (iv) Microevolution occurred in one catheter-related candidemia case. Our results illustrate the relevance of the molecular approach to investigate suspected outbreaks in hospital surveys and the effectiveness of PFGE-Sfi I for typing of epidemiologically related *C. albicans* isolates.

## 1. Introduction

Invasive candidiasis (IC) has substantially increased in neonatal intensive care units (NICUs) over the two past decades and is still associated with a high morbidity and mortality [1, 2]. *Candida albicans* remains the most common causative agent even though non-*albicans* species have been increasingly reported for several years [3]. Acquisition of the *Candida* species by neonates may occur through two different modes: perinatal transmission, mother-neonate (vertical transmission), and nonperinatal transmission, environment-neonate (horizontal transmission) [4]. In hospitalized infants, exogenous origin of *Candida* colonization and infection is well documented. Indeed, many outbreaks of neonatal IC caused by strains originating from hospital staff, biomedical devices, parenteral nutrition, environment, or from other patients have been reported [5–8].

In order to type outbreak-related isolates and to assess their clonality and identify the source and the routes of their transmission, many molecular techniques have been used. They include electrophoretic karyotyping, southern blot hybridization, restriction fragment length polymorphism (RFLP) analysis, randomly amplified polymorphic DNA (RAPD) analysis, PCR-based fingerprinting, and multilocus sequence typing [5, 9–12].

In the NICU of our hospital, six cases of IC due to *C. albicans* were diagnosed within a five-week period. At the same period, two nurses working at the same unit were suffering from *C. albicans* onychomycosis of the fingers. Therefore, a neonatal IC outbreak originating from HCWs strains was suspected. In order to check on this hypothesis, we investigated isolates collected from infected neonates and HCWs at the molecular level by using pulsed-field gel electrophoresis (PFGE) which consisted of electrophoretic karyotyping (EK) and restriction endonuclease analysis of genomic DNA by using Sfi I (PFGE-Sfi I).

## 2. Material and Methods

### 2.1. Patients

Six cases of *C. albicans* IC were identified within a five-week period in the NICU of Farhat Hached University Hospital in Sousse, Tunisia. The NICU consists of one single room with a total of twelve beds. Infected neonates were hospitalized between September 1, 2006 and November 10, 2006. The periods of hospitalization of neonates overlapped and neonates were cared for by the same staff members. Treatment consisted of fluconazole administered intravenously for at least three weeks with removing of the indwelling catheter in all cases. Surgical drainage was used in one neonate with hepatic abscess. The short-term outcome was favorable for five neonates and the remaining neonate died before discharge from the unit. Surveillance program for infection control in the NICU revealed that at the period when the cases occurred, two nurses working at the same unit were harboring *C. albicans* fingers' onychomycosis. Infection-control measures have been reinforced and included rigorous hand-washing in staff members and nurses with onychomycosis were discarded until healing.

Molecular investigations were conducted retrospectively to assess clonality of the *C. albicans* isolates collected during this apparent outbreak.

### 2.2. Isolates

A total of 20 *C. albicans* isolates were typed by EK and PFGE-Sfi I: 18 isolates obtained from the six neonates hospitalized in the NICU and two isolates from onychomycosis of the fingers of two HCWs taking care of the infected neonates.

The sequence of isolates, their anatomical origin, and the time of isolation are summarized in Table 1. The neonates' isolates were collected between September 11 and October 16, 2006. Eleven isolates were collected from blood and deep-site samples, six isolates from implanted medical devices, and one isolate from a urine sample. The number of isolates obtained from a single neonate ranged from one to six isolates. All the neonates have been hospitalized for more than one week prior to the collection of the first isolate. The HCWs' isolates were collected on October 26 and 27, 2006, given that the nurses had nail lesions for several weeks. The ATCC 90028 *C. albicans* reference strain was used as control.

### 2.3. Techniques

#### 2.3.1. Identification of *C. albicans*

The isolates collected from neonates and HCWs were identified as *C. albicans* according to characteristics' growth on Candida ID chromogenic medium (bioMérieux), formation of chlamydospores on potato-carrot-ox gall agar (Bio-Rad), the pattern of sugar assimilation in ID 23C panel (bioMérieux), and the agglutination in the Bichro-albicans test (Fumouze). The isolates were then stored at −80°C in Cryo-billes tubes (Laboratoire AES) until genotyping. Prior to molecular testing, isolates were subcultured on Candida ID medium to assess strain viability and purity.

#### 2.3.2. Preparation of DNA

The preparation of DNA plugs of *C. albicans* was carried out according to the procedure described by Chu et al. with minor modifications [13]. Strains were subcultured onto Sabouraud dextrose agar (SDA) for 48 h at 28°C. Colonies were pelleted by centrifugation at 4000 g for 5 min. The pellet was harvested and washed twice in 1 mL of ET (0.05 mol L^−1^ EDTA, 0.01 mol L^−1^ Tris-HCl, pH 7.5) and resuspended in 150 *μ*L of ET containing 10 U of lyticase (Sigma) and heated to 40°C. Then, 150 *μ*L of 1% agarose (low-melting-point agarose, Promega) in 0.125 mol L^−1^ EDTA (pH 7.5) were added. After mixing, the solution was poured into the wells of, plug moulds and kept at 4°C for 15 min. Upon solidification the agarose plugs were incubated overnight at 37°C in 400 *μ*L of LET (0.5 mol L^−1^ EDTA, 0.01 mol L^−1^ Tris-HCl, pH 7.5) and then transferred in 400 *μ*L of NDS (0.5 mol L^−1^ EDTA, 0.01 mol L^−1^ Tris-HCl, pH 7.5, 1% N-lauroyl sarcosine, 2 mg of proteinase K per mL) for an overnight incubation in a shaker water bath at 50°C. The plugs were washed once with 0.5 mol L^−1^ EDTA (pH 9.0) and stored at 4°C in 0.5 mol L^−1^ EDTA (pH 9.0).

#### 2.3.3. EK Analysis

Chromosomal DNA bands were separated on 1% agarose gels in 0.5x TBE buffer (0.045 mol L^−1^ Tris-HCl, 0.045 mol L^−1^ borate, 1 mol L^−1^ EDTA) in contourclamped homogeneous electric field (CHEF) electrophoresis system (CHEF-DRII, Bio-Rad). Electrophoresis was performed at 150 V for 24 h with a 180 s ramping switch interval and then at 110 V for 22 h with a 360 s ramping switch interval. The temperature of the running buffer was maintained at 14°C.

#### 2.3.4. PFGE-Sfi I Analysis

The plugs were placed in 200 *μ*L of enzyme buffer containing 2 *μ*L of BSA (Promega) and incubated for 1 h at 50°C. The plugs were transferred to 200 *μ*L of enzyme buffer containing 2 *μ*L of BSA and 20 units of Sfi I and incubated at 50°C overnight. Electrophoresis was performed in contour-clamped homogeneous electric field (CHEF) electrophoresis system (CHEF-DRII, Bio-Rad) at pulse time 6–90 s, 180 V in 1% agarose gel for 22 h.

In both molecular techniques, after electrophoresis, the gel was stained with ethidium bromide solution for 15 min and destained with distilled water. A ladder of *Saccharomyces cerevisiae* chromosomal DNA (Bio-Rad) was used as a molecular weight marker.

### 2.4. Clustering Analysis

For both EK and PFGE-Sfi I data analysis, the bands were identified and their size evaluated by using Quantity One 1D analysis software (Bio-rad). The genetic relationships among isolates were established by cluster and ordination analysis performed on the matrix of genetic similaritics. Cluster analysis was performed on the genetic distance matrix with the unweighted paired group method using arithmetic average (UPGMA) and the Jaccard's correlation coefficient calculated on the basis of ERIC-2 patterns by using the MVSP 3.1 software. The Jaccard's coefficient ranges from 1.00 (the two patterns are identical) to 0.00 (no common bands between both patterns). We considered that (i) a Jaccard's coefficient of 1.00 is indicative of strains of the same clone, (ii) a Jaccard's coefficient ranging between 0.90 and 0.99 is indicative of highly similar but nonidentical strains, (iii) a Jaccard's coefficient ranging from 0.80 and 0.89 represents less related isolates, and (iv) a Jaccard's coefficient below 0.79 represents unrelated isolates [14, 15].

## 3. Results

### 3.1. EK Analysis

**Figure 1:**
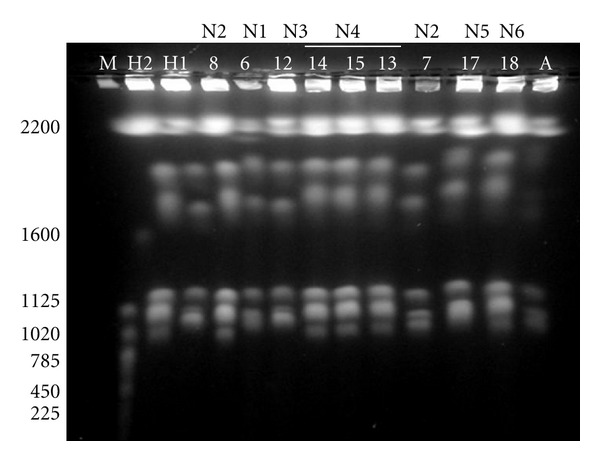
Examples of pulsotypes obtained in EK analysis. M: size marker (in base pairs). H1 and H2: isolates obtained from nurses HCW1 and HCW2, respectively. 6, 12, 17, and 18: isolates obtained from neonates N1, N3, N5, and N6 respectively. 7 and 8: isolates obtained from neonate N2. 13, 14, and 15: isolates obtained from neonate N4. A: ATCC 90028 reference strain.

**Figure 2:**
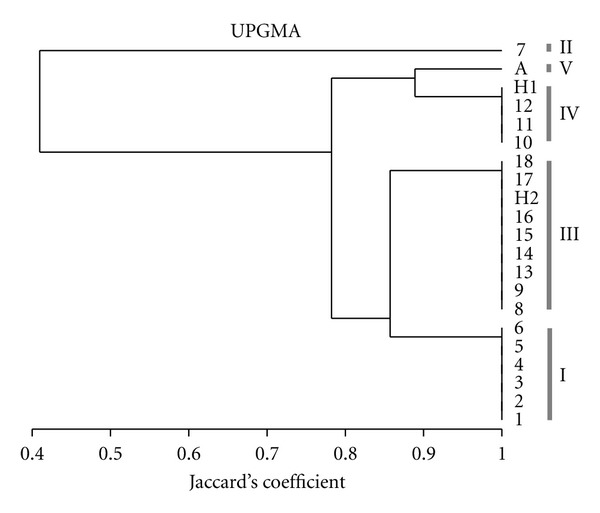
Dendrogram of the genetic relatedness of 20 *C. albicans* isolates from neonates (from 1 to 18) and health care workers (H1 and H2) and ATCC 90028 reference strain (A), based on EK analysis. Karyotypes are noted on right (I to V).

The 20 clinical isolates generated four different karyotypes of six to eight bands each. The karyotypes generated by the HCWs' isolates and nine isolates from neonates are shown in Figure 1. The genetic relatedness of all tested isolates based on EK analysis is illustrated by the dendrogram given in Figure 2. The Jaccard's coefficient values ranged from 0.4 to 1.00. At Jaccard's coefficient of 1.00, four different karyotypes (labeled I to IV) were generated by the clinical isolates. At this level of similarity, isolates grouped into the same cluster are considered as individuals of the same clone. EK analysis allowed identification of three different clones. The ATCC 90028 reference strain (isolate A), used as control, generated a specific karyotype (karyotype V).

### 3.2. PFGE-Sfi I Analysis

**Figure 3:**
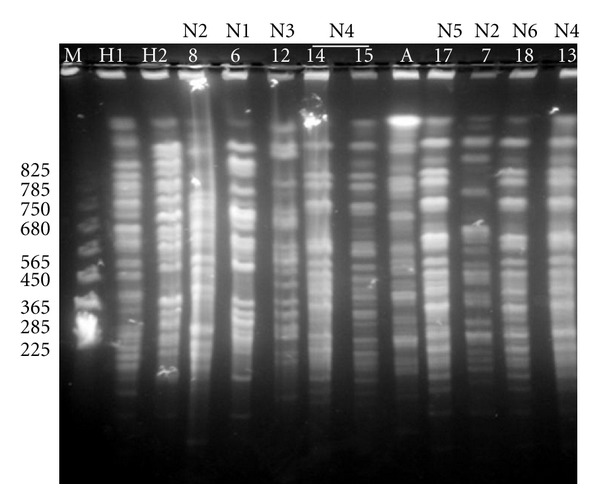
Examples of PFGE patterns of Sfi 1 digested genomic DNA. M: size marker (in base pairs). H1 and H2: isolates obtained from nurses HCW1 and HCW2, respectively. 6, 12, 17, 18: isolates obtained from neonates N1, N3, N5, and N6, respectively. 7 and 8: isolates obtained from neonate N2. 13, 14, and 15: isolates obtained from neonate N4. A: ATCC 90028 reference strain.

**Figure 4:**
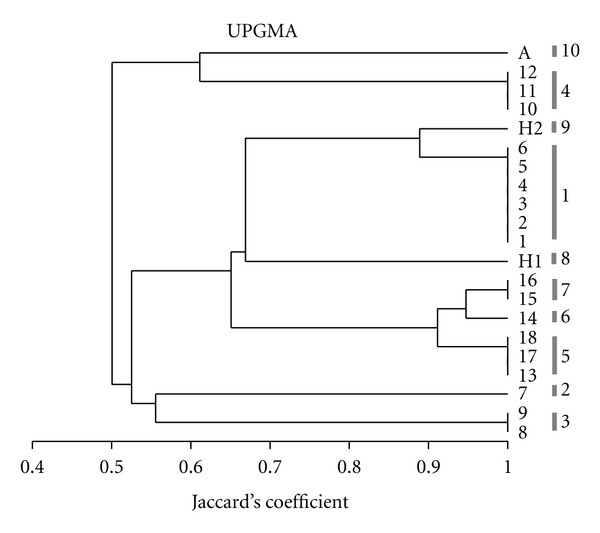
Dendrogram of the genetic relatedness of 20 *C. albicans* isolates from neonates (from 1 to 18) and health care workers (H1 and H2) and ATCC 90028 reference strain (A), based on PFGE-Sfi1 analysis. PFGE-Sfi I patterns are noted on right (1 to 10).

The clinical isolates generated nine different band patterns of 13 to 19 bands each.  Figure 3 shows the PFGE-Sfi I patterns given by the HCWs' isolates and nine isolates obtained from neonates. The molecular relatedness of all tested isolates as determined by PFGE-Sfi I analysis is illustrated by the dendrogram given in Figure 4. The Jaccard's coefficient values ranged from 0.49 to 1.00. At Jaccard's coefficient of 1.00, nine different patterns (labeled 1 to 9) were generated by the clinical isolates and five clones were identified. The ATCC reference strain (isolate A) showed a specific band pattern.

### 3.3. Molecular Relatedness of *C. albicans* Isolates

EK analysis showed that (i) the isolates H1 and H2, obtained from both HCWs one day apart, were unrelated to each other (Jaccard's coefficient of 0.78). (ii) The isolate H1 collected from the HCW1 had an EK pattern identical to three isolates (isolates 10 to 12) collected from the neonate N3. (iii) The isolate H2 collected from the HCW2 yielded an EK pattern identical to eight isolates collected from four neonates (N2, N4, N5, and N6) and was less related (Jaccard's coefficient of 0.86) to the isolates from neonate N1. These findings suggest the occurrence of two outbreaks (karyotypes III and IV) and of cross-contamination between the HCWs and five neonates.

PFGE-Sfi I analysis revealed different epidemiologic features. It showed that (i) the isolate H1 was unrelated to the all isolates collected from neonates (Jaccard's coefficient ≤0.67). (ii) The isolate H2 was less related to the six isolates collected from the neonate N1 (Jaccard's coefficient of 0.88) and unrelated to the other neonates' isolates. (iii) Three isolates (isolates 13, 17, and 18) obtained from neonates N4, N5, and N6 showed the same banding pattern, suggesting that these isolates, collected within a two week-period from the three neonates, were clonal in origin. (iv) Three neonates (N1 to N3) had specific PFGE-Sfi I patterns suggesting coincidental emergence of unrelated strains.

Thus, the PFGE-Sfi I analysis broke the largest EK clone (represented by the karyotype III) into three clones: two clones including each the sequential isolates from the same neonate (N2 and N4) and one clone including isolates from different neonates (N4 to N6).

Of the six neonates included in our study, four had two or more isolates. In two neonates (N1 and N3), sequential isolates from each patient showed the same karyotype and the same PFGE-Sfi I pattern, suggesting that in both N1 and N3 neonates, isolates were clonally related.

In the third neonate (N2), two isolates were collected from blood (isolates 7 and 9) and one isolate from mediastinal fluid (isolate 8). The second blood isolate (isolate 9) and the mediastinal isolate obtained 22 days apart were identical, showing the same karyotype and PFGE-Sfi I pattern, but these two isolates were unrelated to the first blood isolate in both molecular analysis (Table 1, Figures 2 and 4). This suggests strain replacement in the bloodstream infection.

In the fourth neonate (N4), two isolates were collected from blood (isolates 13 and 16) and two umbilical catheter tips (isolates 14 and 15). Interestingly, PFGE-Sfi I analysis showed that the first blood isolate (isolate 13) and the first catheter isolate (isolate 14), collected one week later, were highly similar to each other, exhibiting minor band differences (Jaccard's coefficient of 0.92). The second catheter isolate (isolate 15) and the second blood isolate (isolate16) collected 11 days later were identical to each other and highly similar to the isolate 14 (Jaccard's coefficient of 0.95). These findings suggest that the four isolates derived from the same strain and this strain underwent two successive microevolutions.

Microevolutionary changes were not detected by EK analysis where the four isolates showed the same karyotype.

It is important to note that, according to the PFGE-Sfi I analysis, the first blood isolate (isolate 13) of the neonate N3 was identical to the isolates 14 and 15, collected from the neonates N4 and N5, suggesting that the isolates from the three neonates derived from the same strain which underwent microevolution in only neonate N4.

## 4. Discussion

Nosocomial candidiasis has been increasing over the last decades and is still associated with a high morbidity and mortality rates despite the availability of novel antifungal agents [1, 2]. This underlines the importance of rational prevention measures. For such purpose, knowledge of a local epidemiology of IC in NICU settings is much needed. Various molecular typing methods are now available that allow understanding of the *Candida* spp. transmission, colonization and infection in neonatal care setting [5, 9–12]. It has long been assumed that the vertical transmission of the *Candida* species, mainly *C. albicans*, is an important mode of acquisition of the yeasts by neonates at delivery [4]. However, many reports showed that hospitalized infants more frequently acquire *C. albicans* from the hospital environment than from their mothers [16, 17]. In addition, several studies emphasized the role of hands of HCWs in horizontal transmission [5–7, 9]. Indeed, the exposure of neonates to HCWs' strains was associated with outbreaks of neonatal IC, and catheters and parenteral nutrition were shown to be involved as routes of contamination [18]. It is worth mentioning that most HCWs involved in nosocomial transmission of Candida species in neonatal setting were asymptomatic carriers [5–7, 9, 18]. 

At first insight, the temporal clustering in our NICU of six cases of neonatal IC caused by *C. albicans* and the concomitant occurrence of nails' onychomycosis in HCWs, due to the same species at the same period, argue for the likeliness of a neonatal IC outbreak with HCWs as the source. In order to check on this hypothesis, we investigated isolates collected from infected neonates and HCWs by EK and PFGE-Sfi I. Our results showed that, overall, there was no agreement between results of both typing methods and that PFGE-Sfi I better discriminated among isolates of *C. albicans* as compared to EK analysis. Indeed, we identified nine different PFGE-Sfi I patterns but only four different karyotypes. PFGE-Sfi I analysis was able to differentiate isolates with the same karyotype. However, the reverse was not seen as different karyotypes were always associated with different PFGE-Sfi I patterns. This finding is consistent with the results of many previous studies [19, 20]. Therefore, in order to investigate the outbreak, only the PFGE-Sfi I findings were taken into account. The isolates H1 and H2 collected from the HCWs were unrelated to each other and unrelated to the neonates' isolates ruling out the likeliness that the HCWs' strains caused infections in neonates. Of the six neonates included in our study, three had specific strains which disproves the hypothesis that the three cases were correlated to the investigated outbreak. However, the fact that the cases were coincidental does not rule out their nosocomial origin. Indeed, nosocomial IC has been shown to arise either as an outbreak or sporadic cases [21]. 

The remaining N4 to N6 three neonates were infected by the same strain, as showed by identical PFGE-Sfi I patterns. As the three neonates occupied the same NICU at the same time, there is evidence supporting the horizontal transmission of *C. albicans* in the unit, whether by cross-contamination between neonates or exposure of neonates to a common source. Unfortunately, because of the retrospective nature of our study, additional samples from potentially colonized HCWs and from unit environment were not available. We are, therefore, unable to further identify the source and the mode of transmission of *C. albicans* during the outbreak. Nevertheless, the isolation of the same strain from the umbilical catheters of two neonates suggests that the outbreak was subsequent to the extrinsic contamination of catheters. In addition, the occurrence a of single strain shared by different patients, as in our three neonates, argues for its nosocomial origin and warrants reinforcing and implementing further control measures in order to reduce the extent of the outbreak [22]. 

Many previous studies showed that *C. albicans* strains when sequentially isolated from the same patient are most often identical whatever they are isolated from the same sites or from different noncontiguous sites [23, 24]. This finding is explained by the predominant clonal reproduction and the lack of a sexual reproduction in *C. albicans *[25]. Occasionally, different strains may coexist in the same patient [23, 24]. Furthermore, it is currently admitted that *C. albicans* infection is mainly endogenous, caused by colonizing strains. In addition, minor genetic changes in sequences of individual DNA fragments occur sporadically in commensal and infecting isolates [24, 26]. Such genetic variations occurring at the site of colonization and through recurrent episodes of infection are commonly called microevolution [24–26]. 

In our study, the sequential isolates were identical in both neonates N1 and N3. In neonate N1, isolates collected from three different body sites and from two medical devices over the 24-day period were identical and did not showe any genetic variation in both used molecular analyses. In neonate N3, three isolates were sequentially collected from urine, blood, and umbilical catheter, two and five days apart respectively. In this case, because the catheter was removed as soon as the blood culture was shown to be positive and because catheter colonization frequently results in catheter-related blood stream infection [27], we assume that, in this neonate, the blood and urine isolates arose recently from the catheter. 

In neonate N4, the isolates recovered from the catheters were identical or highly similar to the blood isolates. This suggests that the portal of entry of the yeast was the catheter and that the four isolates derived from the same strain which underwent microevolution. It is worth mentioning that two successive microevolutionary changes occurred in the neonate N4. The same strain infected two additional neonates (N5 and N6) in whom only a single isolate was collected, which precludes checking the stability of the strain in both infants. These findings confirm the occurrence of microevolutionary changes in catheter-related candidemia but do not allow estimating its frequency because of the small number of cases investigated in our study. Shin et al. showed that microevolution occurred in 36% of catheter strains when compared to blood strains [19]. Approximately, the same frequency (33%) was reported by Marco et al. in colonizing strains (urine, stool, and other sites) [28]. 

In neonate N4, microevolution could only be detected by PFGE-Sfi I, whereas, in EK analysis, all isolates were identical to each other and to isolates collected from the neonates N5 and N6. This finding confirms that PFGE-Sfi I method is a powerful means of detecting microevolution among sequential isolates of *C. albicans* as a compared to EK analysis. It is in accordance with previous reports and indicates that microevolution may occur without a change in the size of the *C. albicans* chromosomes [19, 20]. 

In neonate N2, both blood isolates, collected approximately one month apart, showed different karyotypes and different PFGE-Sfi I patterns and were widely separated in both dendrograms. This finding suggests strain replacement in blood as change in the karyotype of serial clinical isolates of *C. albicans* was shown to be indicative of the occurrence of a new strain [19, 29]. The coexistence of two different strains is unlikely because of the long time that separated the isolation of both strains. 

In conclusion, our results showed that only three out of the six investigated *C. albicans* IC cases could be correlated to the outbreak and preclude the hypothesis that the HCWs were the source. This illustrates the importance of the molecular approach to investigate suspected outbreaks in hospital survey. The confirmation of the spread of a unique strain prompts further control measures to reduce the extent of the outbreak. In addition, our findings confirm that the PFGE-Sfi I is effective both for cluster analyses of temporally related *C. albicans* isolates and for detecting microevolution within individual patient isolates as compared with the EK method. 

As a limited number of isolates were investigated, larger studies on *C. albicans* infection and colonization are warranted in order to better assess the horizontal transmission in the acquisition of *Candida* species by neonates in our unit. 

## Figures and Tables

**Table 1:** Description of the 20 *Candida albicans* isolates investigated and summary of results of EK and PFGE-Sfi I analysis.

Neonate or HCW*	Isolate	Site of isolation	EK pulsotype	PFGE-Sfi I pattern	Date of sampling (day/mo/yr)	Time of hospitalisation
N1	1	Blood	I	1	11/09/2006	
	2	Endotracheal tube	I	1	13/09/2006	
	3	Postoperative wound	I	1	26/09/2006	Admission: 01/09/2006
	4	Hepatic abscess	I	1	27/09/2006	Discharge: 07/11/2006
	5	Drain	I	1	04/10/2006	
	6	Postoperative wound	I	1	04/10/2006	

N2	7	Blood	II	2	15/09/2006	Admission: 06/09/2006
	8	Mediastinal fluid	III	3	19/09/2006	Discharge: 08/11/2006
	9	Blood	III	3	11/10/2006	

N3	10	Urine	IV	4	26/09/2006	
	11	Blood	IV	4	29/09/2006	Admission: 16/09/2006
	12	Umbilical catheter	IV	4	05/10/2006	Discharge: 08/11/2006

N4	13	Blood	III	5	29/09/2006	
	14	Umbilical catheter	III	6	05/10/2006	Admission: 20/09/2006
	15	Umbilical catheter	III	7	16/10/2006	Death: 16/10/2006
	16	Blood	III	7	16/10/2006	

N5	17	Blood	III	5	13/10/2006	Admission: 05/10/2006 Discharge: 10/11/2006

N6	18	Umbilical catheter	III	5	04/10/2006	Admission: 21/09/2006 Discharge: 21/10/2006

HCW1	H1	Fingers's nails	IV	8	26/10/2006	—

HCW2	H2	Fingers's nails	III	9	27/10/2006	—

ATCC 90028	A		V	10	—	—

*Health care worker.
